# Rubber Compounds from Devulcanized Ground Tire Rubber: Recipe Formulation and Characterization

**DOI:** 10.3390/polym16040455

**Published:** 2024-02-06

**Authors:** Ákos Görbe, Andrea Kohári, Tamás Bárány

**Affiliations:** 1 Department of Polymer Engineering, Faculty of Mechanical Engineering, Budapest University of Technology and Economics, Műegyetem rkp. 3., H-1111 Budapest, Hungary; gorbea@pt.bme.hu (Á.G.); koharia@pt.bme.hu (A.K.); 2 PolymerOn Ltd., Háros u. 7., H-1222 Budapest, Hungary; 3 MTA-BME Lendület Lightweight Polymer Composites Research Group, Műegyetem rkp. 3., H-1111 Budapest, Hungary

**Keywords:** ground tire rubber, GTR, rubber compound, vulcanization properties, rubber recycling, devulcanization, soybean oil, circular economy

## Abstract

In this study, our focus was on developing and investigating rubber recipes that are suitable for devulcanized ground tire rubber (dGTR). Devulcanized rubber has a powdery or sticky uncured rubber-like appearance depending on the extent of main-chain degradation that occurs with selective crosslinking scission. Still, it has a significantly shorter scorch time than a new rubber compound. Therefore, our primary goal was to slow down the vulcanization process of dGTR and improve its mechanical properties via recipe development. We formulated several recipes (sulfur-, peroxide-, and phenolic resin-based) and studied the vulcanization process and the main properties of the revulcanized rubber sheets. We observed that the vulcanization process could be altered with different vulcanization methods: using peroxide and vulcanizing resin extended the process significantly. Peroxide vulcanization also provided enhanced elongation compared to sulfuric systems. With a balance of properties in mind, we selected a semi-efficient sulfur-based recipe and studied the characteristics of natural rubber/dGTR mixtures with the help of plasticizer oils. We successfully replaced a notable portion of natural rubber with dGTR, maintaining its properties without much compromise.

## 1. Introduction

The recycling of elastomers proves to be one of the most critical problems related to recycling [1]. This difficulty is due to the structure of elastomers: their high elasticity is provided by crosslinks between their molecule chains. These crosslinks, however, make them hard to recycle as they hinder reversible melting. It makes them impossible to recycle, unlike thermoplastic polymers, which are characterized by reversible meltability. The most pressing issue with elastomer recycling is recycling tire rubber. Tire rubbers are composite materials consisting of metallic and textile reinforcements and different types of rubber. First, the elastomeric part must be ground, making ground tire rubber (GTR). This GTR is easy to handle, and it can be used to toughen different kinds of polymers, for example, thermoplastics [2,3,4,5], thermosets [6,7], and rubbers as well [8,9,10,11]. The problem with this kind of use is that the compatibility of GTR with the desired matrix is usually poor, hindering high performance.

These solutions, while viable, cannot be considered optimal as they cannot handle the increasing mass of rubber waste. A more optimal way of recycling is devulcanization and reclamation, both of which aim to break up the crosslinked structure of rubbers, thus enabling primary recycling [12]. Devulcanization achieves this goal by selective crosslink scission; reclamation is accompanied by chain scission [13]. There are several ways of devulcanization; the most widespread methods are thermomechanical [14,15,16], thermochemical [17,18] and microwave devulcanization [19,20].

The most valid solution is thermomechanical devulcanization because it is continuous, highly productive, and does not involve harmful chemical agents [15]. Thermomechanical devulcanization is carried out mainly in a twin-screw extruder, where combining heat and shear produces enough energy to break crosslinks between chains [21]. Heat and shear can be altered easily in the extruder as their screws are modular and thus easy to configure. Thermomechanical devulcanization can be aided with chemical additives that help break crosslinks. Supercritical carbon dioxide (scCO_2_) is considered a green devulcanizing additive and is gaining attention in the rubber industry. The main effect of scCO_2_ is that it swells the GTR and amplifies the scission of crosslinks [22,23].

The degree of devulcanization can be rated by soluble content and crosslink density. Horikx’s analysis establishes a mathematical relationship between the sol fraction and the decrease in crosslink density, and it can be used to characterize devulcanization [14,24,25].

The main obstacle for devulcanizates is that if they are revulcanized, their properties are inferior to those of primary rubbers. Researchers have also found that the accelerator residue remaining in the dGTR after devulcanization significantly reduces the scorch time of the compounds during revulcanization [26]. This fast revulcanization is also emphasized by residual carbon black because of the oxygen, nitrogen, and sulfur on its surface [27]. Fast revulcanization might also result from devulcanization itself, as reduced crosslinking density increases polymeric chain mobility, leading to more effective collisions among molecules and a more rapid revulcanization reaction [28].

The idea of a circular economy is present in all parts of the rubber industry: beyond the spread of recycled rubber, bio-based plasticizers are recognized for their excellent properties and renewable nature [29]. The source of these plasticizers can be either recycled plastic or rubber via pyrolysis [30,31], or vegetable oil, like palm oil, soybean oil, castor oil, sunflower oil, and linseed oil. Vegetable oils are the most common types of bio-based plasticizers; they are characterized by unsaturated fatty acids, and they are used to improve the flowability of rubber compounds [30,31]. They are also used in other industries, mainly for synthesizing new polymers [32,33]. Soybean oil (SBO) is highly promising in this area since it is inexpensive and its properties can be tailored [34,35]. They are mainly used in silica-based rubber compounds and have been found to improve the compatibility of the phases through their unsaturated bonds [36]. A similar effect has also been found in carbon-black-based compounds [37].

Our goal in this study was to investigate different vulcanization recipes on dGTR to enhance mechanical properties and decelerate the revulcanization. We also aimed to examine the effect of replacing half of the virgin natural rubber in the recipe with dGTR. We also studied the possibility of replacing synthetic oils with soybean oil from renewable sources. The novelty of our work comes from the utilization of renewable soybean oil in recycled rubber compounds and its comparison with regular mineral oil, which has not been studied in depth before.

## 2. Materials and Methods

### 2.1. Materials

Devulcanized ground tire rubber (dGTR) was kindly provided by Tyromer Inc. (Waterloo, ON, Canada). Ground tire rubber, made from truck tires with a particle size under 1 mm, is thermomechanically devulcanized in an extruder with the help of scCO_2_. The company also provided the original GTR they used during the process. We used a general-purpose natural rubber (NR), SMR CV 60 (Akrochem Corp; Akron, OH, USA; Mooney viscosity (ML, 1 + 4, 100 °C): 55–65). We used different additives in the rubber compounds listed in Table 1. The recipes can be found in Section 2.2.

### 2.2. Preparation of dGTR-Based Rubber Compounds

We prepared the rubber compounds using a Brabender Lab-Station internal mixer (Brabender GmbH & Co. KG (Duisburg, Germany)) equipped with a W 350 E chamber (free volume 370 cm^3^). The temperature was set to 50 °C, and the batches were mixed at 40 rpm. The exact recipes and their contents can be found in Table 2. We examined three types of curatives: sulfuric, peroxidic, and phenolic ones. We formulated nine different recipes combining different accelerators and co-activators. This way, we were able to study the effects of a conventional (Sulfur3), an efficient (Sulfur5) and a semi-efficient (Sulfur2) system. We also changed the amount of dGTR in different recipes. Tire rubber contains a considerable amount of carbon black, fillers (~30%) and other additives (~10%) other than rubber, which remain in the dGTR after devulcanization. Therefore, we added 167 phr of dGTR to the recipes to ensure that the rubber part is approximately 100 phr. The amount of activators stayed the same during the experiment, as we aimed to examine the effect of the accelerators and crosslinking agents. We selected the quantities used according to our previous experience and the manufacturers’ recommendations.

We also prepared compounds with NR and two different processing oils: soybean oil and aromatic oil (Table 3). We investigated the impact of replacing a substantial portion (50%) of natural rubber with dGTR within a rubber compound. We formulated these recipes based on the results of the first round of experiments with the addition of carbon black and two types of processing oils. The selected sulfuric recipe from the first round was altered (mainly the amount and rate of accelerators) to facilitate slower curing and a stronger resulting crosslink network.

Based on our previous research [19], we used the so-called two-step mixing. This means that in the first step, we added vulcanizing agents to the dGTR, and in the second step, we added the premixes (dGTR_mix_, dGTR_soybean_, and dGTR_aromatic_) to the NR-based rubber compounds.

We prepared 2 mm thick rubber sheets of the compounds with a Teach-Line Platen Press 200E hot press (Dr. Collin GmbH (München, Germany)) at 180 °C and 200 bar. The pressing time was determined according to the vulcanization times (t_90_) of each compound. We used standardized dyes to punch the specimens from these sheets.

### 2.3. Characterization Methods

We used thermogravimetric analysis (TGA) to characterize the composition and thermal behavior of GTR and dGTR. We carried out the experiment on 10 mg samples using a TGA Q500 machine (TA Instruments Ltd., New Castle, DE, USA). The analysis was performed from room temperature to 800 °C with a heating rate of 10 °C/min in a nitrogen atmosphere, with a gas flow of 60 mL/min.

We characterized the dGTR using Horikx’s analysis [14]. Horikx’s analysis is a method that establishes a relationship between the soluble content and the decrease in crosslink density after devulcanization. The soluble fraction of the dGTR samples was determined by Soxhlet extraction in toluene, and the decrease in crosslink density was determined with swelling.

We studied the curing properties of the rubber compounds with a MonTech D-RPA 3000 (MonTech (Buchen, Germany)) rubber process analyzer. We characterized the vulcanization at 180 °C (1.67 Hz and 1° amplitude) for 30 min and calculated the main parameters of the vulcanization process. Scorch time (t_10_) was calculated as the time to reach 10% cure and vulcanization time (t_90_) was calculated as the time to reach 90% cure. The cure rate index (CRI) was calculated with Equation (1).

(1) $$CRI=\frac{100}{t_{90}-t_{10}}$$

We also determined the difference between the maximum (S′_max_) and minimum (S′_min_) of the torque curve, which can be used to infer the change in the crosslink density of the sample.

The crosslink density of the samples was determined using the equilibrium swelling test method. The samples were soaked in toluene for 72 h at room temperature. After that, the samples were removed from the solvent, dried with paper towels, and the swollen mass was measured. The samples were then dried at 80 °C for 12 h and their mass was measured again.

We calculated the crosslink density using the Flory–Rehner Equation (2).

(2) $$\nu_{e}=\frac{-\left[ln(1-V_{r}\right]+V_{r}+\chi {\cdot V}_{r}^{2}]}{\left[V_{s}\cdot \left(V_{r}^{\frac{1}{3}}-\frac{V_{r}}{2}\right)\right]}$$

where ν_e_ is crosslink density (mol/cm^3^), V_s_ is the molar volume of the solvent (for toluene: 106.27 cm^3^/mol), χ is the Flory–Huggins interaction parameter (0.391), and V_r_ is the volume fraction of rubber in the swollen network. V_r_ can be calculated using the Ellis–Welding Equation (3).

(3) $$V_{r}=\frac{\frac{m_{r}}{\rho_{r}}}{\frac{m_{r}}{\rho_{r}}+\frac{m_{s}}{\rho_{s}}}$$

where m_r_ is the weight of the dry sample (g), m_s_ is the weight of the solvent absorbed by the sample (g), ρ_r_ is the density of the rubber sample (g/cm^3^), and ρ_s_ is the density of the solvent (for toluene: 0.867 g/cm^3^) [38,39].

We also determined the swelling index (%) of the samples using Equation (4).

(4) $$Swellingindex=\frac{m_{sr}-m_{r}}{m_{r}}$$

where m_sr_ is the weight of the swollen sample (g).

The density of the samples was determined according to the ASTM D 297-93 standard [40] (hydrostatic method) with a Sartorius Quintix 125D semi-micro balance with a resolution of 0.01 mg. The test medium was distilled water with a temperature of 20.8 °C and a corresponding density of 0.998 g/cm^3^.

The viscoelastic properties of the rubber compounds were analyzed using a MonTech D-RPA 3000 (MonTech (Buchen, Germany)) rubber process analyzer by oscillating shear. A frequency sweep was performed on the specimens at 30 °C between 0.1 and 100 Hz (at least 5 measurements at each level). The amplitude was chosen previously according to an amplitude sweep at 10 Hz at 30 °C from 0.01° to 1.5°. We determined the border of linear viscoelasticity based on 30 measuring points at every amplitude and chose an adequate amplitude for the frequency sweep.

We performed tensile (according to ISO 37 [41], Type 2 dumbbell specimens) and tear tests (according to ISO 34 [42], Method B, angle test pieces) on standardized specimens to measure the basic mechanical properties of the rubber compounds. A Zwick Z005 (Zwick GmbH (Ulm, Germany)) universal testing machine equipped with a 5 kN load cell was used with a crosshead speed of 500 mm/min for both tests.

We measured the hardness of the samples according to the ISO 48-4 [43] Shore A method with a Zwick H04.3150.000 type hardness tester (Zwick GmbH, Ulm, Germany). Indentation time was 3 s, and the load was 8.05 N.

## 3. Results

### 3.1. Characterization of dGTR

**Figure 1:**
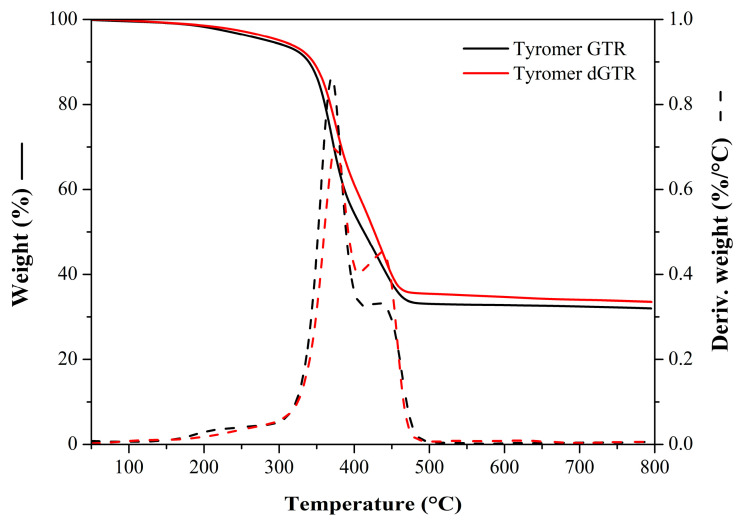
TGA and DTG curves of GTR and dGTR.

The TGA curves of GTR and dGTR (Figure 1) show that the thermal properties did not change considerably during devulcanization. However, it is apparent from the DTG curves that some changes occurred in the molecular structure, as the peaks differ slightly from the curve representing GTR. This change can be attributed to changes in crosslink density and chain scission.

We determined the composition of the GTR using its TGA curve and the decomposition temperature of the component. We assumed that the GTR contains roughly ~5% additives, ~45% natural rubber, ~17% synthetic rubber, and ~33% carbon black and other inorganic fillers. This composition agrees with a typical recipe for truck tire tread rubbers.

We analyzed the dGTR using Horikx’s analysis and found that the decrease in crosslink density was moderate (~60%). However, the sol fraction was relatively high (~30%). This proves the main effect was rather random chain scission than selective crosslink scission. Random chain scission reduced molecular weight and, in turn, provided the dGTR with a more malleable nature.

### 3.2. The Effect of Different Vulcanization Systems

#### 3.2.1. Cure Characteristics

**Figure 2:**
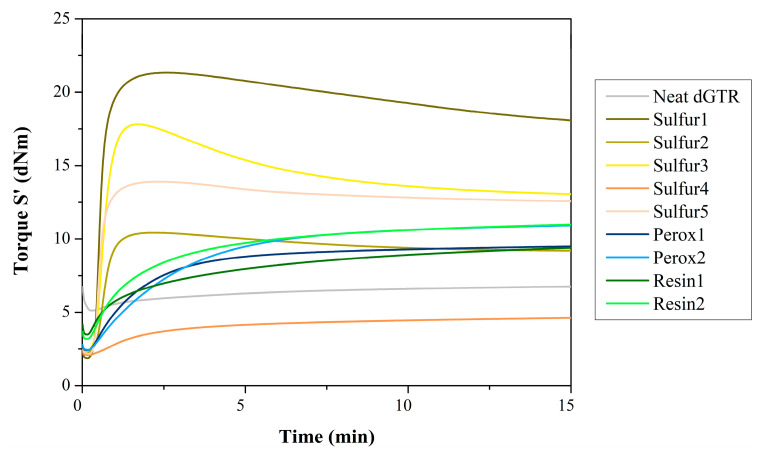
Vulcanization curves of the rubber compounds.

We characterized the vulcanization of the rubber compounds prepared with different recipes (Figure 2, Table 4). The compound containing 100 phr dGTR (Sulfur1) had a higher maximum torque and a faster vulcanization time than the ones with 167 phr dGTR. This is due to the increase in accelerators compared to other recipes. Sulfur1 contained less rubber than other recipes, resulting in higher crosslink density, which yielded higher torques and faster vulcanization.

The different compositions did not produce a significant change in vulcanization time (t_90_) in the case of the sulfuric systems; nearly all compounds had a t_90_ around 1 min. Also, the recipe without primary vulcanization agents (not counting the sulfur in CBS) (Sulfur 4) showed signs of vulcanization. One explanation might be that the remaining sulfur present in the dGTR acts as a residual vulcanizing agent. Another possible explanation could be recombination, which supposedly occurs during devulcanization when the sulfur bridges break up. When this breakup happens, the resulting half-bridges can react with each other at the elevated temperature of devulcanization. When this reaction occurs between half-bridges, they can recombine, forming new bridges. These are fewer in number, but they make the devulcanization incomplete. The unbound half-bridges can account for the rapid vulcanization during revulcanization. This effect can be observed on the vulcanization curves; the neat dGTR also shows signs of vulcanization without any vulcanizing agent or accelerators.

In the case of peroxidic curing, we slowed down the reaction considerably while producing higher torques compared to the recipe with no crosslinking agents. This is evident from the significantly lower CRI values. This is in connection with the crosslinking mechanism of peroxides: they produce carbon–carbon links between chains by decomposing at high temperatures. These links are not in connection with sulfur. Thus, the residue in dGTR does not interfere with the crosslinking process. Vulcanization curves (Figure 2) show that peroxide-cured compounds are less susceptible to reversion than the sulfur-cured compounds. This effect is due to the stronger and more stable nature of carbon–carbon links compared to sulfur bridges. We also noted that using DIPP peroxide allows for higher torques and slower vulcanization times than DCP.

We studied the vulcanization process using resorcinol-based vulcanization resins. These materials are mainly used as adhesives that promote crosslinking, thus improving adhesion to fabric and steel cord. This type of vulcanization yielded the slowest vulcanization times without reversion in the corresponding curves. However, the measured torques were relatively low compared to other compounds, indicating a weaker crosslink network. This is confirmed by the fact that the sheets remained sticky after vulcanization and did not retain their shape; they were deformed.

#### 3.2.2. Crosslink Density

We determined the densities (ρ_r_) of the samples to calculate their crosslink density. The results (Table 5) show that the different vulcanization systems had no significant effect on the density of the rubber samples.

**Figure 3:**
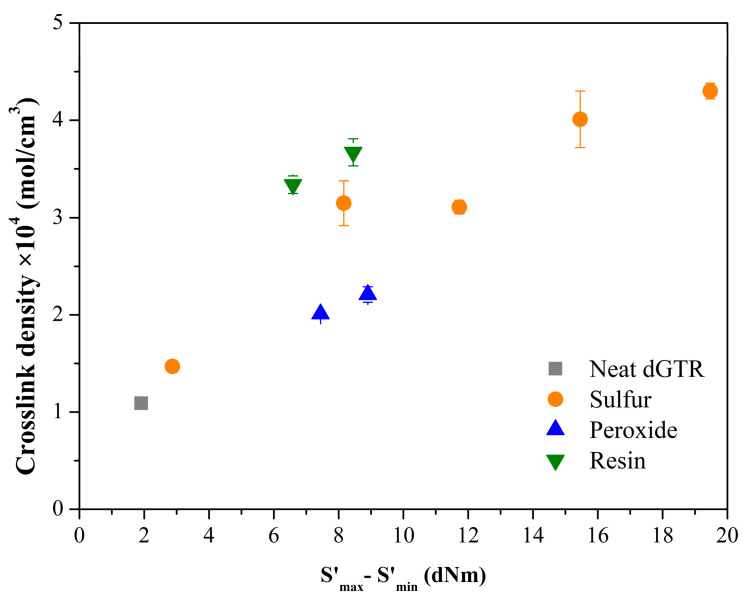
Correlation of crosslink density and S′_max_ − S′_min_.

The crosslink density values (Table 5) of the samples are in good agreement with those observed during the investigation of the vulcanization characteristics (Figure 3) [44]. As expected, neat dGTR had the lowest crosslink density, while a slight increase was observed with the activator and accelerator (Sulfur4). Increasing the amount of sulfur increased the amount of crosslinks in the samples. Peroxide-based compounds (Perox1 and Perox2) had a lower crosslink density. For the resin-based compounds (Resin1 and Resin2), a similar crosslink density was measured as for the sulfur-based ones. This contradicts our previous experience. After drying the swollen specimens, we found that these samples became stiffer than the original material or even the other compounds. This is probably due to some chemical processes caused by toluene in the samples. Further measurements are needed to prove this.

#### 3.2.3. Dynamic Properties

**Figure 4:**
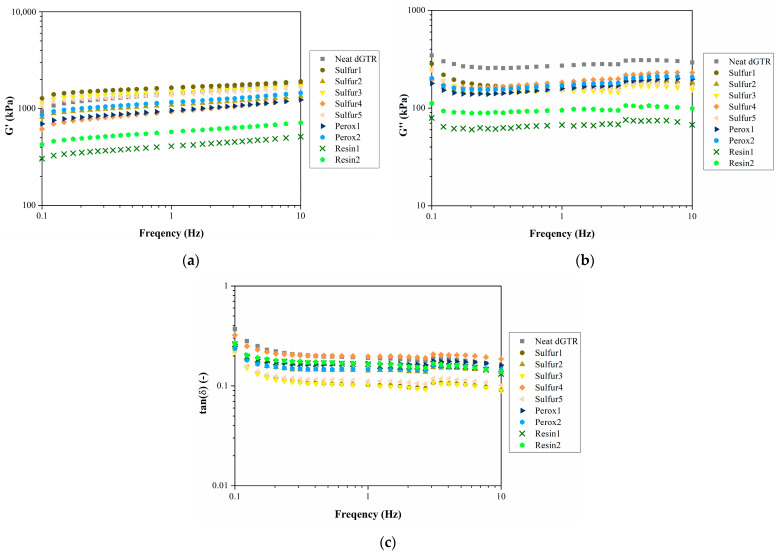
Viscoelastic properties of dGTR compounds: (**a**) storage modulus (G′); (**b**) loss modulus (G″); (**c**) damping (tan(δ)).

We performed DMTA analysis to determine the viscoelastic properties of the dGTR-based compounds (Figure 4). We found that their dynamic properties are closely connected with their crosslink density. This can be observed best from the damping properties, as specimens with higher crosslink density (e.g., Sulfur1, Sulfur3, and Sulfur5) exhibit slightly worse damping than specimens with lower crosslink density. Increasing the crosslink density makes the material stiffer, resulting in a reduced damping effect. As a result, neat dGTR and Sulfur4 exhibit the best damping since their crosslink density is the lowest of the specimens.

As for the peroxidic systems, there are no significant differences in damping; the compound with the higher crosslink density (Perox2) shows smaller damping. The resin systems are close to each other, resulting from their almost identical crosslink density.

#### 3.2.4. Hardness

The hardness of the rubber compounds (Table 6) shows that increasing the dGTR content softens the material (100 to 167 phr dGTR). A conventional system (Sulfur3) resulted in an even lower hardness than the semi-efficient (Sulfur2) system due to the resulting elastic polysulfide crosslinks. Without incorporating any sulfur, the compound without sulfur (Sulfur4) was the softest. The efficient system (Sulfur5) was slightly harder than the conventional one due to the more stable and stiffer di- and monosulfide crosslinks. Due to the carbon–carbon crosslinks, the peroxide-cured systems (Perox1 and Perox2) had decreased hardness compared to sulfuric recipes. The phenolic resin-cured systems had similar hardness as sulfuric systems. However, they were far stickier compared to other compounds and prone to tear during vulcanization, so we decided not to investigate the phenolic resin-cured system further.

#### 3.2.5. Tensile Tests

**Figure 5:**
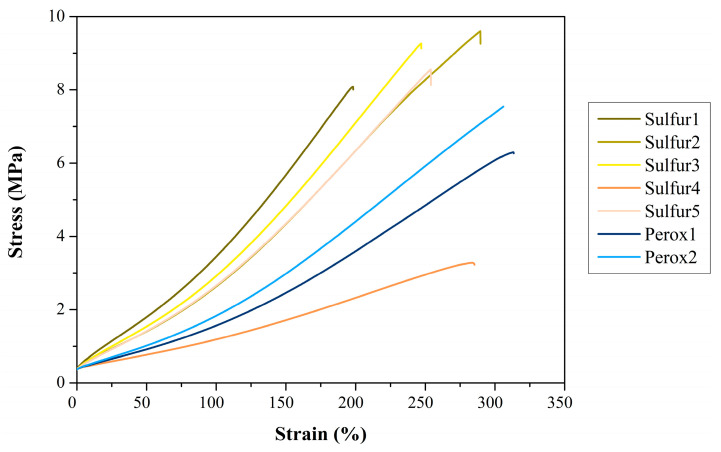
Typical tensile curves of the dGTR-based compound.

The tensile test results (Figure 5, Table 7) showed that the recipes yielded quite different results, especially strain at break, which was mostly influenced. A higher amount of dGTR did not have a significant impact on tensile strength; the results are overlapping. However, we increased strain at break significantly due to the increased specific rubber content and decreased filler content, which made the rubber compound more elastic because of the lower crosslink density. We also found that increased sulfur content enhanced tensile strength and moduli while reducing strain at break compared to the semi-efficient system. If there was no sulfur in the recipe, the resulting vulcanizate was rather soft; it had low tensile strength and the relatively high strain at break. It was caused by the low crosslink density. Combining different accelerators does not produce a stiffer material; adding TMTD to the recipe did not result in better mechanical properties. This may be because the reduced amount of sulfur was not able to create a strong, elastic crosslink network.

The peroxidic vulcanization systems provided the rubber compounds with the highest strain at break. This is due to the stronger carbon–carbon crosslinks, which can withstand higher elongation without breaking. However, the expected rise in tensile strength compared to sulfuric recipes did not occur. This is in connection with the recipes; more peroxide might have raised tensile strength. The difference between DCP and DIPP was evident: DIPP produced a stiffer material with higher strength and lower strain at break. This may be in connection with thermal stability: DIPP is characterized by a higher content of active oxygen, which makes it more reactive than DCP. That resulted in a higher crosslink density with stiffer behavior.

#### 3.2.6. Tear Test

**Figure 6:**
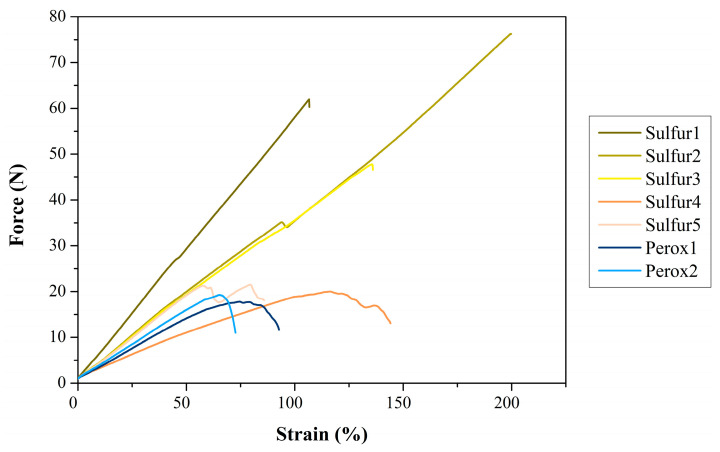
Typical tear curves of the dGTR-based compound.

The tear tests (Figure 6, Table 7) are used to determine the resistance against crack propagation. We found that the increase in dGTR content from 100 (Sulfur1) to 167 phr (Sulfur2) in the recipe improved tear strength and strain significantly. This can be due to the change in the ratio of residual accelerators and the vulcanization system, which yielded a lower crosslink density. Increasing sulfur does not provide higher tear strength compared to the semi-efficient system. This may be in connection with the formation of polysulfide crosslinks, which are prone to high reversion at high temperatures (Figure 2). These crosslinks are more elastic than mono- or disulfide crosslinks, which is also apparent in this case. The absence of sulfur (Sulfur4) resulted in a rather soft material with low resistance against cracking. The efficient system (Sulfur5) is characterized by lower tear strength than other sulfuric systems. The resulting monosulfidic bonds also make for low strain.

The peroxidic recipes behaved similarly to each other, and they both fell short of sulfuric systems. This behavior may be in connection with the vulcanizing mechanism of peroxides: they break up chains by decomposition, and this breakup makes the material vulnerable to crack propagation.

### 3.3. Recipes with Additional Uncured Virgin NR and Oils

#### 3.3.1. Cure Characteristics

**Figure 7:**
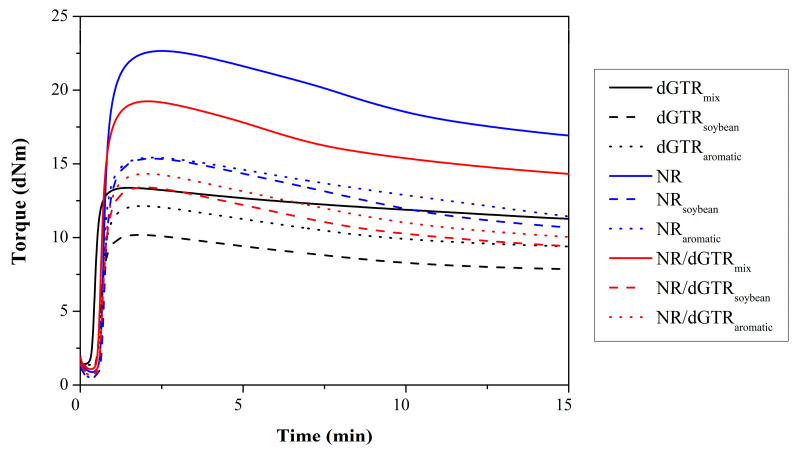
Curing curves of the rubber compounds with additional NR and oil.

We formulated the second round of recipes with the modification of the Sulfur5 recipe from the first series. We increased the ratio of sulfur in the recipe to achieve better mechanical properties, mainly elongation. The cure characteristics (Figure 7, Table 8) of the compounds show that the dGTR-based compound was much faster than the NR without dGTR because of the residual accelerators. We also found that the compound of NR and dGTR resulted in a slower curing rate than dGTR, with usually higher torques. It can also be seen that the addition of NR increased sensitivity against heat, as a higher reversion is detectable on the curves.

We found that the incorporation of oils into the dGTR compounds successfully decelerated the process in case of dGTR-based compounds and, of course, reduced the torques. Soybean oil (SBO) is known to take part in the vulcanization process because of its unsaturated nature. It means that it can take up the remaining accelerator and sulfur; this way, it can slow the process down. Aromatic oil, on the other hand, is a highly reactive and nonpolar oil that can also bind free radicals and residual accelerators, hindering vulcanization [45]. In the case of NR-based compounds, the incorporation of oils reduced sensitivity against heat; reversion was decreased significantly.

#### 3.3.2. Crosslink Density

We determined the densities (ρ_r_) of the samples to calculate their crosslink density. The results (Table 9) show that NR-based compounds have the lowest density. Compounds containing dGTR have a slightly higher density due to the styrene–butadiene rubber content of the ground tire rubber.

**Figure 8:**
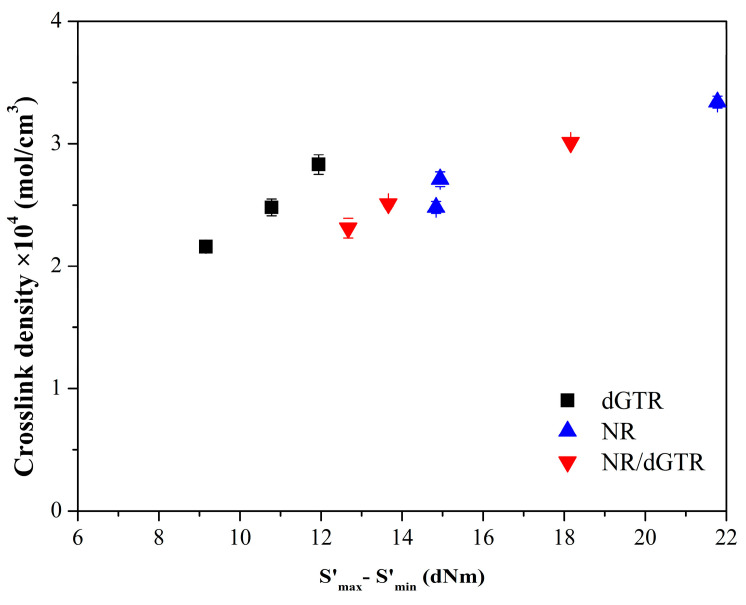
Correlation of crosslink density and S′_max_ − S′_min_.

The measured crosslink density values are consistent with the S′_max_ − S′_min_ values (Figure 8). The oils in the compounds reduced their crosslink density. This reducing effect was more significant for soybean oil. This is probably because soybean oil contains unsaturated bonds that can bind sulfur, so less of it can be involved in crosslinking the rubber.

#### 3.3.3. Dynamic Properties

**Figure 9:**
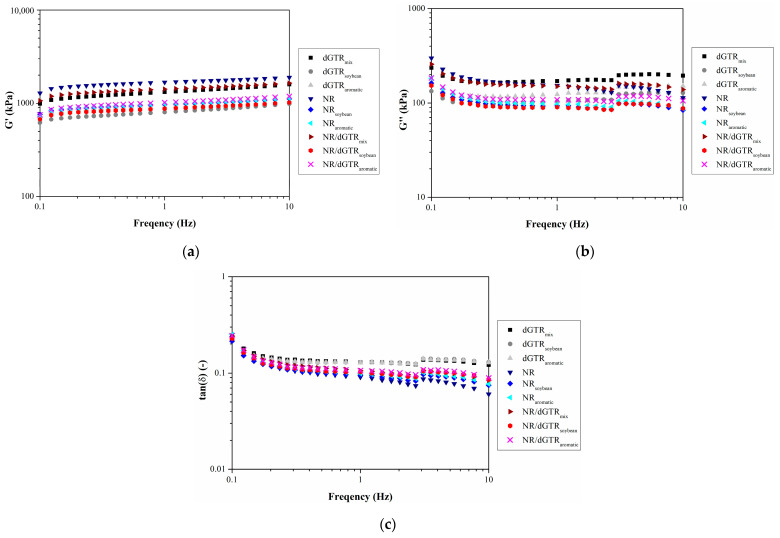
Viscoelastic properties of NR-based compounds: (**a**) storage modulus (G′); (**b**) loss modulus (G″); (**c**) damping (tan(δ)).

We also studied the viscoelastic properties of the NR-based compounds (Figure 9). We established a similar connection between crosslink density and damping as in the first round: higher crosslink density produces a stiffer material characterized by lower damping. This means that NR has the worst damping, and dGTR is characterized by relatively high damping because of its lower crosslink density. The NR/dGTR mix is between the two components in this regard.

The effect of processing oil is not apparent on the damping of the dGTR compounds, but the storage and loss moduli were reduced significantly. In the case of NR compounds, damping was improved due to the incorporation of processing oils; they also altered crosslink density significantly. The NR/dGTR mixes are similar to the dGTR compounds: damping did not change significantly, unlike the storage and loss moduli.

#### 3.3.4. Hardness

The hardness of the rubber sheets (Table 10) shows that both types of oils softened the material significantly. We observed a more pronounced plasticizing effect in the case of SBO. This can be associated with the lower viscosity of SBO compared to aromatic oil; that way, it was able to diffuse into the molecules, allowing for a softer behavior. Another explanation might be that SBO, as an unsaturated oil, is prone to use up sulfur, and this way, we achieved lower crosslink density than aromatic oil.

It can also be seen that dGTR-based compounds are much softer compared to NR-based compounds. This is caused by the degradation of molecules, which is inevitable during devulcanization.

#### 3.3.5. Tensile Test

**Figure 10:**
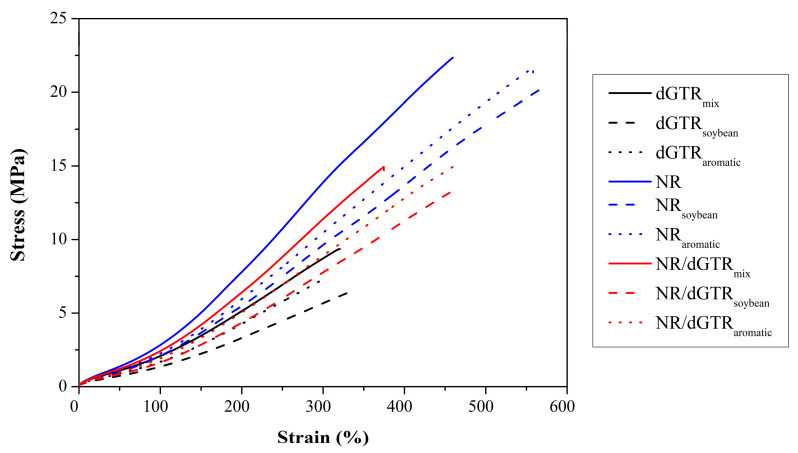
Typical stress–strain curves for the rubber compounds with additional NR and oil.

The results of the tensile tests (Figure 10, Table 11) show that modifying the Sulfur5 recipe from the first series of experiments resulted in higher tensile strength and elongation at break. This indicates that the modified ratio of accelerators and vulcanizing agents produced a more stable crosslink network. We also observed that while SBO improved strain, the aromatic oil did not have such an effect. This may be because soybean oil is a highly unsaturated vegetable oil, making it prone to covulcanization with rubber. Thus, it can behave as a coupling agent between the rubber molecules and carbon black. Additionally, SBO is highly unsaturated, which makes it prone to vulcanization. This can result in covulcanization, where SBO reacts with sulfur and forms a stronger crosslink network [46]. Additionally, the polar nature of SBO may have aided in the better dispersion of polar carbon black in the nonpolar rubber matrix, resulting in fewer aggregates acting as starting points for cracks.

Similar behavior was observed in the case of NR compounds; unsaturated soybean oil seemed to promote better adhesion between the molecules and carbon black.

The NR/dGTR compounds were characterized as intermediate between neat NR and dGTR compounds. The adhesion between the rubbers was adequate, as we enhanced both strain and tensile strength compared to neat dGTR.

#### 3.3.6. Tear Test

**Figure 11:**
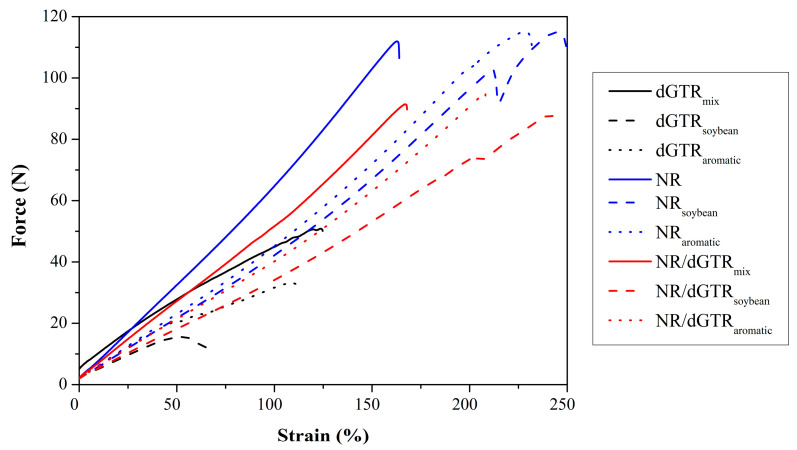
Typical tear curves of the rubber compounds with additional NR and oil.

The tear tests (Figure 11, Table 11) indicate that the oils prompted a different kind of response in dGTR compared to tensile tests: both tear force and strain decreased significantly. This may be caused by the fact that shorter molecular chains in dGTR are more susceptible to crack propagation compared to neat NR, and this susceptibility was only enhanced by the incorporated oil.

In the case of neat NR and NR/dGTR compounds, additional oil enhanced the strain but did not significantly decrease the tear force, thanks to the strong crosslink network formed by the active softeners.

We concluded that substituting NR with dGTR did not hinder tear strength significantly because of the good adhesion between the dGTR and NR phases. When the dGTR phase breaks, the NR phase can still hold the specimen together due to its large molecular weight.

## 4. Conclusions

We prepared several recipes based on commercial devulcanized ground tire rubber and investigated their main features. The recipes we formulated contained a conventional, an efficient, and a semi-efficient sulfuric system; two types of peroxides and two types of phenol resins were used. The conventional recipe resulted in more polysulfide links, which made the material elastic, and the semi-efficient and efficient systems created di- and monosulfide links, which improved tensile properties.

The peroxide curing resulted in lower vulcanization because they did not react strongly to residual sulfur in the dGTR. These systems also enhanced strain at break and reduced reversion compared to sulfuric systems, but they also had lower tensile strength. We also experienced similar results with the resin systems.

We studied the amount of dGTR in recipes and found that when filler residues are taken into account, the resulting rubber becomes more elastic and softer. This is related to higher specific rubber content and crosslink density. We also established a correlation between crosslink density and damping; we found that adding dGTR to an NR recipe significantly improves its damping ability.

We formulated rubber blends by replacing 50% of natural rubber with dGTR, observing a minimal worsening of vulcanization, tensile, and tear properties. Among the processing oils studied, soybean oil showed superior performance. With it, we produced high-quality, greener vulcanizates using recycled GTR and processing oil from renewable sources. We believe our work is an important step towards inserting rubber waste into the circular economy alongside soybean oil and creating more sustainable rubber compounds that can be used for high-quality applications (for example, tires).

## Figures and Tables

**Table 1:** Types, producers, trademarks, and functions of raw materials used in the rubber compounds.

Material	Manufacturer	Trademark	Function
ZnO zinc oxide	Werco Metal (Zlatna, Romania)	-	Activator
stearic acid	Oleon (Ertvelde, Belgium)	Radiacid 0154	
CBS N-cyclohexyl-2-benzothiazolesulfenamide	Rhein Chemie (Mannheim, Germany	Rhenogran CBS-80	Accelerator
TMTD tetramethylthiuram disulfide	Lanxess (Mannheim, Germany)	Rhenogran TMTD-70	Accelerator
sulfur	Ningbo Actmix Polymer (Ningbo, Zhejiang, China)	ACTMIX S-80	Curing agent
DCP dicumyl peroxide	Norac (Azusa, CA, USA)	Norox DCP-40BK	Curing agent
DIPP di-(2-tert.-butyl-peroxyisopropyl)-benzene	Nouryon Pergan GmbH (Bocholt, Germany)	Peroxan BIB-40 EV-G	Curing agent
BDMA 1,4-butanediol dimethacrylate	Lanxess (Mannheim, Germany)	Rhenofit BDMA/S	Coagent
HMMM hexa(methoxymethyl)melamine ether	Rhein Chemie (Mannheim, Germany)	Cohedur A 250	Curing agent
resorcinol	Rhein Chemie (Mannheim, Germany)	Cohedur RS	Curing agent
resorcinol	Rhein Chemie (Mannheim, Germany)	Rhenogran Resorcin-80	Curing agent
silica	Lanxess (Mannheim, Germany)	Vulkasil C	Catalyst
aromatic oil	Klaus Dahleke KG (Hamburg, Germany)	Tudalen 4353	Processing oil
soybean oil	Vandamme Hungary Ltd. (Komárom, Hungary)	Degummed non GMO	Processing oil
N550 carbon black	Omsk Carbon Group (Omsk, Russia)	-	Filler

**Table 2:** The recipes of the rubber compounds.

	Amount of Ingredients (phr)								
	Sulfur1	Sulfur2	Sulfur3	Sulfur4	Sulfur5	Perox1	Perox2	Resin1	Resin2
dGTR	100	167	167	167	167	167	167	167	167
ZnO	5	5	5	5	5	-	-	-	-
stearic acid	2	2	2	2	2	-	-	-	-
CBS	1.5	1.5	1.5	3	1	-	-	-	-
TMTD	-	-	-	-	1	-	-	-	-
sulfur	1.5	1.5	3	-	1	-	-	-	-
DCP	-	-	-	-	-	2	-	-	-
DIPP	-	-	-	-	-	-	2	-	-
BDMA	-	-	-	-	-	0.5	0.5	-	-
Cohedur A 250	-	-	-	-	-	-	-	4.6	4.8
Cohedur RS	-	-	-	-	-	-	-	3.4	-
Rhenogran Resorcin-80	-	-	-	-	-	-	-	-	3
Vulkasil C	-	-	-	-	-	-	-	15	15.8

**Table 3:** The recipes of the rubber compounds containing NR and oil.

	Amount of Ingredients (phr)								
	dGTR_mix_	NR	NR/dGTR_mix_	dGTR_soybean_	NR_soybean_	NR/dGTR_soybean_	dGTR_aromatic_	NR_aromatic_	NR/dGTR_aromatic_
dGTR	167	-	-	167	-	-	167	-	-
NR CV60	-	100	100	-	100	100	-	100	100
dGTR_mix_	-	-	100	-	-	-	-	-	-
dGTR_soybean_	-	-	-	-	-	100	-	-	-
dGTR_aromatic_	-	-	-	-	-	-	-	-	100
Soybean oil	-	-	-	10	10	10	-	-	-
Aromatic oil	-	-	-	-	-	-	10	10	10
ZnO	5	5	5	5	5	5	5	5	5
Stearic acid	2	2	2	2	2	2	2	2	2
Carbon black	-	50	50	-	50	50	-	50	50
CBS	1.5	1.5	1.5	1.5	1.5	1.5	1.5	1.5	1.5
TMTD	0.5	0.5	0.5	0.5	0.5	0.5	0.5	0.5	0.5
Sulfur	1	1	1	1	1	1	1	1	1

**Table 4:** Vulcanization characteristics of the rubber compounds.

Compound	S′_min_ (dNm)	S′_max_ (dNm)	S′_max_ − S′_min_ (dNm)	t_10_ (min)	t_90_ (min)	CRI (min^−1^)
Neat dGTR	5.12	7.03	1.91	0.61	18.83	5.49
Sulfur1	1.87	21.34	19.47	0.39	0.95	178.57
Sulfur2	2.26	10.42	8.16	0.43	1.06	158.73
Sulfur3	2.35	17.81	15.46	0.41	1.01	166.67
Sulfur4	2.08	4.95	2.87	0.57	16.62	6.23
Sulfur5	2.17	13.90	11.73	0.39	0.90	196.08
Perox1	2.41	9.85	7.44	0.45	7.32	14.56
Perox2	2.44	11.34	8.90	0.56	8.58	12.47
Resin1	3.48	10.07	6.59	0.35	15.26	6.71
Resin2	3.19	11.64	8.45	0.41	12.06	8.58

**Table 5:** The density and crosslink density of the revulcanized samples.

Compound	Density, ρ_r_ (g/cm^3^)	Swelling Index (%)	Crosslink Density, ν_e_ (mol/cm^3^·10^−4^)
Neat dGTR	1.163 ± 0.003	346.3 ± 6.9	1.09 ± 0.04
Sulfur1	1.166 ± 0.003	163.6 ± 1.7	4.30 ± 0.08
Sulfur2	1.171 ± 0.002	194.8 ± 8.1	3.15 ± 0.23
Sulfur3	1.158 ± 0.017	171.8 ± 9.7	4.01 ± 0.29
Sulfur4	1.148 ± 0.010	299.8 ± 2.7	1.47 ± 0.02
Sulfur5	1.162 ± 0.007	197.5 ± 2.5	3.11 ± 0.07
Perox1	1.126 ± 0.006	257.6 ± 0.7	2.01 ± 0.01
Perox2	1.154 ± 0.010	240.6 ± 5.0	2.21 ± 0.08
Resin1	1.160 ± 0.008	189.8 ± 2.9	3.34 ± 0.09
Resin2	1.158 ± 0.004	180.2 ± 3.9	3.67 ± 0.14

**Table 6:** Shore A hardness of the revulcanized samples.

Compound	Hardness (ShA°)
Sulfur1	59.9 ± 0.5
Sulfur2	52.0 ± 1.6
Sulfur3	49.3 ± 0.8
Sulfur4	34.4 ± 0.7
Sulfur5	51.2 ± 1.8
Perox1	38.7 ± 0.7
Perox2	42.5 ± 2.1
Resin1	53.8 ± 0.8
Resin2	52.9 ± 2.7

**Table 7:** Results of the tensile and tear tests.

Compound	Tensile Strength (MPa)	Strain at Break (%)	M100 (MPa)	M200 (MPa)	Tear Strength (N/mm)
Sulfur1	8.1 ± 0.5	199.2 ± 4.6	2.58 ± 1.72	-	29.3 ± 0.8
Sulfur2	8.6 ± 0.7	282.6 ± 21.6	2.43 ± 0.18	5.77 ± 0.52	37.2 ± 1.5
Sulfur3	9.0 ± 0.9	264.9 ± 34.9	2.73 ± 0.23	6.57 ± 0.54	20.7 ± 4.1
Sulfur4	3.2 ± 0.2	285.7 ± 7.9	1.18 ± 0.01	2.31 ± 0.01	6.5 ± 0.5
Sulfur5	8.0 ± 0.4	258.3 ± 11.7	2.50 ± 0.14	5.60 ± 0.33	12.4 ± 2.9
Perox1	6.1 ± 0.2	314.8 ± 4.4	1.53 ± 0.05	3.50 ± 0.13	5.9 ± 0.4
Perox2	7.8 ± 0.2	306.5 ± 31.8	1.91 ± 0.24	4.62 ± 0.64	5.6 ± 0.9

**Table 8:** Vulcanization times and torques of the rubber compounds with additional NR and oil.

Sample	S′_min_ (dNm)	S′_max_ (dNm)	S′_max_ − S′_min_ (dNm)	t_10_ (min)	t_90_ (min)	CRI (min^−1^)
dGTR_mix_	1.43	13.37	11.94	0.35	0.65	333.33
dGTR_soybean_	1.02	10.18	9.16	0.5	0.9	250.00
dGTR_aromatic_	1.36	12.14	10.78	0.54	0.96	238.10
NR	0.88	22.66	21.78	0.62	1.12	200.00
NR_soybean_	0.52	15.36	14.84	0.64	1.08	227.27
NR_aromatic_	0.49	15.43	14.94	0.58	1.01	232.56
NR/dGTR_mix_	1.08	19.24	18.16	0.54	0.98	227.27
NR/dGTR_soybean_	0.74	13.41	12.67	0.59	1.01	238.10
NR/dGTR_aromatic_	0.67	14.33	13.66	0.59	1.02	232.56

**Table 9:** The density and crosslink density of the samples with additional NR and oil.

Compound	Density, ρ_r_ (g/cm^3^)	Swelling Index (%)	Crosslink Density, ν_e_ (mol/cm^3^·10^−4^)
dGTR_mix_	1.149 ± 0.002	210.0 ± 3.3	2.83 ± 0.08
dGTR_soybean_	1.142 ± 0.003	245.1 ± 2.5	2.16 ± 0.04
dGTR_aromatic_	1.142 ± 0.006	227.1 ± 3.7	2.48 ± 0.07
NR	1.127 ± 0.008	195.3 ± 1.5	3.34 ± 0.05
NR_soybean_	1.118 ± 0.008	232.0 ± 2.6	2.48 ± 0.05
NR_aromatic_	1.118 ± 0.008	221.2 ± 2.6	2.71 ± 0.06
NR/dGTR_mix_	1.120 ± 0.004	208.4 ± 0.7	3.01 ± 0.02
NR/dGTR_soybean_	1.136 ± 0.002	237.8 ± 4.7	2.31 ± 0.08
NR/dGTR_aromatic_	1.125 ± 0.004	229.1 ± 1.1	2.51 ± 0.02

**Table 10:** The hardness of the rubber compounds with additional NR and oil.

Compound	Hardness (ShA°)
dGTR_mix_	53.5 ± 0.8
dGTR_soybean_	43.7 ± 0.4
dGTR_aromatic_	48.0 ± 1.0
NR	59.5 ± 0.4
NR_soybean_	47.2 ± 1.1
NR_aromatic_	51.3 ± 0.9
NR/dGTR_mix_	54.4 ± 0.6
NR/dGTR_soybean_	44.6 ± 1.0
NR/dGTR_aromatic_	48.7 ± 0.5

**Table 11:** Results of the tensile and tear tests of the rubber compounds with additional NR and oil.

Sample	Tensile Strength (MPa)	Strain at Break (%)	M100 (MPa)	M200 (MPa)	Tear Strength (N/mm)
dGTR_mix_	9.27 ± 0.31	320.9 ± 17.1	2.05 ± 0.07	5.05 ± 0.16	24.9 ± 5.0
dGTR_soybean_	6.56 ± 0.32	340.8 ± 13.9	1.35 ± 0.02	3.29 ± 0.03	8.2 ± 0.4
dGTR_aromatic_	7.34 ± 0.55	306.6 ± 17.5	1.68 ± 0.04	4.22 ± 0.10	17.4 ± 3.2
NR	21.27 ± 1.00	454.3 ± 20.3	2.82 ± 0.02	7.60 ± 0.22	57.7 ± 2.4
NR_soybean_	19.48 ± 1.02	570.3 ± 22.1	1.93 ± 0.11	5.05 ± 0.28	57.0 ± 5.1
NR_aromatic_	20.80 ± 0.90	550.9 ± 18.6	2.12 ± 0.17	5.63 ± 0.39	57.0 ± 6.4
NR/dGTR_mix_	15.00 ± 0.17	371.6 ± 5.9	2.47 ± 0.04	6.55 ± 0.11	48.7 ± 7.6
NR/dGTRs_oybean_	13.22 ± 0.69	461.1 ± 20.4	1.59 ± 0.05	4.22 ± 0.12	43.4 ± 3.1
NR/dGTR_aromatic_	15.52 ± 0.40	463.7 ± 7.2	1.91 ± 0.04	5.08 ± 0.09	40.6 ± 6.3

## Data Availability

Data are contained within the article.
